# Use of a Modified Transpalatal Arch to Correct a Severely Rotated Maxillary Incisor in the Mixed Dentition: A Case Report

**DOI:** 10.7759/cureus.97199

**Published:** 2025-11-19

**Authors:** Jed Lee, Robert Smyth

**Affiliations:** 1 Department of Orthodontics, Eastman Dental Hospital, University College London Hospitals NHS Foundation Trust, London, GBR

**Keywords:** interceptive orthodontics, maxillary incisor rotation, mesiodens, modified transpalatal arch, supernumerary teeth

## Abstract

An erupted mesiodens can cause displacement and rotation of the maxillary incisors, which may impact a child's smile aesthetics and their confidence. This case report presents a non-compliance method for correcting maxillary incisor rotations in the mixed dentition using a modified transpalatal arch in combination with a sectional fixed appliance. The patient was a nine-year-old male who presented in the mixed dentition with a Class I incisor relationship on a Class I skeletal base with an erupted mesiodens, upper arch crowding, and severe (110°) rotation of the right maxillary central incisor. De-rotation of the incisor was accomplished within 17 weeks with preservation of periodontal health and gingival aesthetics. The clinical significance of this case report is that the modified transpalatal arch offers a simple and compliance-free approach for correcting severe maxillary incisor rotations and is a practical alternative to removable appliances in the mixed dentition.

## Introduction

A tooth rotation is defined as the intra-alveolar displacement of a tooth around its longitudinal axis and may arise from a range of pre- or post-eruptive disturbances [1,2]. The anterior maxillary teeth, commonly referred to as the ‘social six’, are the most visible components of a person’s smile, and their normal eruption and alignment are fundamental to facial aesthetics [3]. As such, eruptive disturbances involving the maxillary incisors can have a substantial impact on a child’s psychosocial development, thereby warranting timely interceptive orthodontic management [4].

Teeth rotations are most effectively corrected using force couple mechanics, which is defined as the application of two forces equal in magnitude but opposite in direction separated by a perpendicular distance [5]. Force couples can be generated in a variety of ways using fixed orthodontic appliances and auxiliaries, removable appliances, or a combination of both [6]. Correction of maxillary incisor rotations in the mixed dentition is particularly challenging because only a limited number of teeth are available for bonding and anchorage. As a result, removable appliances are often used for anchorage control. However, treatment success with removable appliances relies on excellent patient compliance, which can be difficult to achieve in younger children. Temporary anchorage devices (TADs) such as orthodontic mini-screws offer a compliance-free alternative, but their use in young children can be problematic due to the presence of the developing permanent dentition, which reduces the availability of safe and stable insertion sites [7,8]. Therefore, this case report demonstrates the use of a modified transpalatal arch (TPA) as an alternative non-compliance method for correcting maxillary incisor rotations in the mixed dentition.

## Case presentation

Patient information

This case report was prepared following the CARE Guidelines [9]. The patient was a medically fit 9-year-old male referred by a primary care orthodontist to the Orthodontic Department at Eastman Dental Hospital for an erupted mesiodens that had caused severe rotation of the upper right central incisor (UR1). According to the patient’s mother, the mesiodens had erupted several years earlier, but no treatment was advised by the general dentist at the time or during subsequent dental recall appointments. As the UR1 rotation worsened over time, the family sought a second opinion from another general dentist, who subsequently referred the patient to the local orthodontist. The patient’s main concern was the poor aesthetics of the rotated UR1, which affected his confidence due to the negative remarks he frequently received about his appearance from peers at school.

Diagnostic assessment

Extra-Oral Assessment

**Figure 1:**
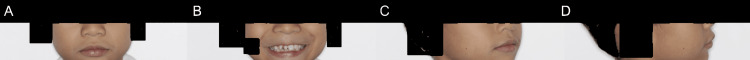
Pre-treatment extra-oral photographs. (A) Frontal view, (B) frontal smiling view, (C) off-center frontal view, and (D) lateral view.

The patient presented with a Class I skeletal profile with average vertical dimensions and no facial asymmetry. He had a normal smile line, but his smile aesthetics were poor due to the erupted mesiodens and displaced UR1 (Figure 1).

Intra-Oral Assessment

**Figure 2:**
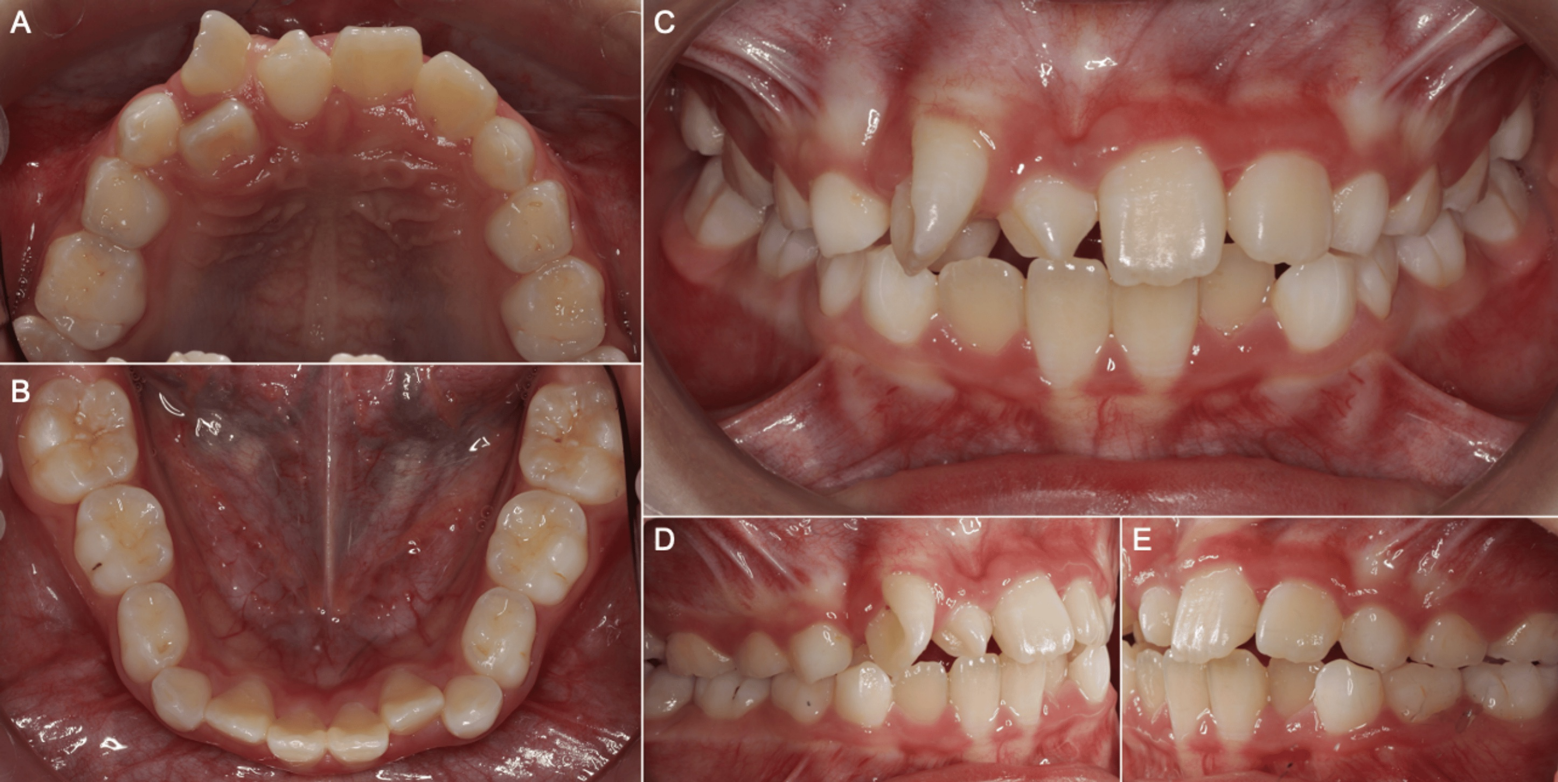
Pre-treatment intra-oral photographs. (A) Upper arch, (B) lower arch, (C) anterior occlusion, (D) right occlusion, (E) left occlusion.

The patient was in the mixed dentition with 8 mm maxillary arch crowding due to an erupted mesiodens. The UR1 was displaced distally, severely rotated 110º mesio-palatally, and incompletely transposed with the palatally displaced upper right lateral incisor (UR2) (Figure 2). The mandibular arch was mildly crowded in the labial segment.

In occlusion, the incisor and molar relationships were Class I. The upper centerline, assessed from the mesial surface of the upper left central incisor (UL1), was coincident with the facial midline and lower centerline. The UR2 was in crossbite with the lower right lateral incisor (LR2) and deciduous canine (LRC) with no displacement.

Radiographic Assessment

**Figure 3:**
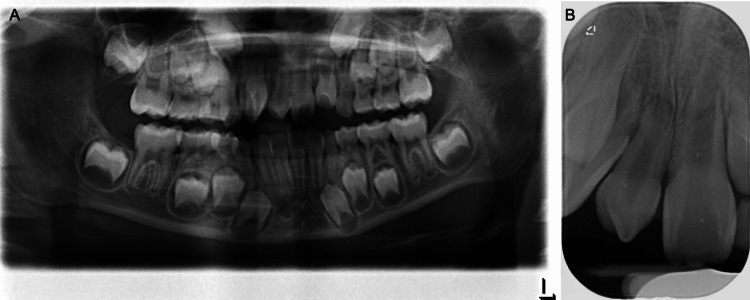
Pre-treatment radiographs. (A) Dental panoramic radiograph, (B) periapical radiograph.

The dental panoramic radiograph showed the presence of all permanent teeth, excluding third molars, and the presence of a supernumerary tooth in the maxillary midline region (Figure 3). A periapical radiograph of this area showed that the supernumerary had an immature, conical root. 

Diagnosis

The patient was a 9-year-old male with a Class I malocclusion in the mixed dentition, presenting with an erupted mesiodens, 8 mm of upper arch crowding, severe (110°) mesio-palatal rotation of the UR1, and palatal displacement of the UR2.

Etiology

The etiology of the patient’s malocclusion was the erupted mesiodens, which caused a tooth size-arch length discrepancy in the maxillary arch. This resultant crowding led to severe rotation of the UR1 and displacement of both the UR1 and UR2.

Intervention

Treatment aimed to improve the patient’s smile aesthetics by aligning the UR1. This was important because the malocclusion had already begun to have a negative psychosocial impact on the patient.

The treatment plan involved (1) a referral to the patient’s general dentist for extraction of the mesiodens and maxillary primary canines for space creation, (2) interceptive orthodontic treatment to align the UR1 using a modified transpalatal arch and an upper sectional fixed appliance with 0.022” x 0.028” Damon^TM^ Q2 self-ligating brackets (Ormco Corporation, Glendora, CA), and (3) retention with an 0.0195” stainless steel (SS) lab-made upper palatal fixed retainer and a removable thermoformed Theroux retainer.

**Figure 4:**
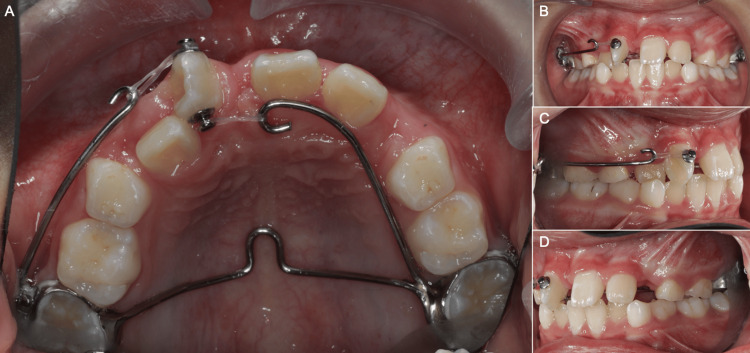
Design of the modified transpalatal arch with two hooks soldered to the molar bands on the first permanent molars. (A) Occlusal view, (B) frontal view, (C) right view, (D) left view.

The movements required to align the tooth were de-rotation initially, followed by mesial bodily movement. To de-rotate the UR1, a modified TPA was constructed with two distal-facing hooks, a buccal hook on the right and a palatal hook on the left, made of 1 mm SS soldered to molar bands and cemented to the maxillary first permanent molars using glass ionomer cement (GIC) (Ketac™ Cem Radiopaque Glass Ionomer Luting Cement, 3M Deutschland GmbH, Germany) (Figure 4). Round base bondable buttons (DB Orthodontics Ltd., Orpington, UK) were attached to the mesial and distal surfaces of UR1 using light-cured composite (Transbond™ XT Light Cure Adhesive Paste, 3M Unitek, Monrovia, CA), ensuring that the buttons and the hooks were all in the same horizontal plane to avoid unwanted vertical force vectors. Elastomeric powerchain (Dura-chain, Ortho-Care Ltd, Bradford, UK) was attached from the distal button to the buccal hook on the right, and from the mesial button to the palatal hook on the left. The elastomeric chains were activated by gentle stretching to initial force levels of approximately 150 g. This configuration generated an initial force couple (moment) of approximately 1200 g·mm (150 g × 8 mm) in the mesio-labial direction. These force levels were expected to reduce over time as elastomeric chains have been shown to undergo force decay of up to 70% over 28 days [10]. 

As the UR1 de-rotated, the hooks were manually shortened to allow continued stretch and activation of the elastomeric powerchain. This was achieved by removing the modified TPA, cutting the hooks using a Maun cutter, and reforming the hooks using orthodontic pliers before recementing the modified TPA with GIC. Once sufficient de-rotation was achieved (at 13 weeks), a sectional fixed appliance was bonded to the upper central incisors (UR1, UL1), upper left lateral incisor (UL2), and upper right deciduous first molar (URD) using Damon Q2 passive self-ligating brackets. These brackets were selected due to their ability to ensure full ligation of the archwires. The URD was bonded to reduce the inter-bracket distance and flexibility of the archwire on the right side, thereby reducing the risk of disengagement of the archwire from the molar tube. The mesial and distal buttons were removed to avoid interference with alignment, and a button was bonded palatally to allow continuation of force couple mechanics. The force couple was maintained through engagement of a 0.012” nickel-titanium (NiTi) archwire on the labial side and an elastomeric power chain from the palatal button to the left palatal hook of the modified TPA.

**Figure 5:**
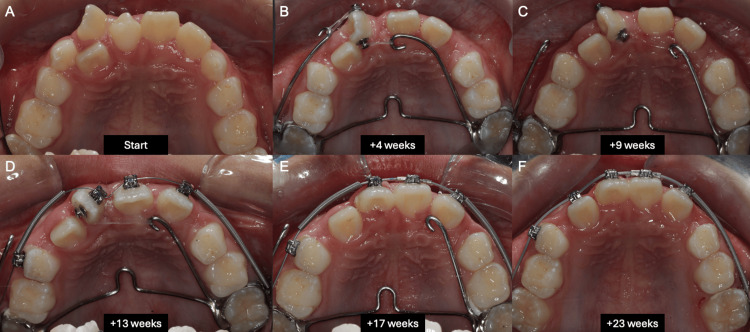
Treatment progress over 23 weeks. (A) Pre-treatment, (B) at 4 weeks, (C) at 9 weeks, (D) at 13 weeks, (E) at 17 weeks, and (F) at 23 weeks.

Complete de-rotation of UR1 was achieved at the following visit (17 weeks) (Figure 5). Table 1 summarizes the de-rotation progress of UR1 over time. The UR2 had moved labially spontaneously as the UR1 aligned, and this tooth was included in the fixed appliance at 23 weeks. The modified transpalatal arch was also removed at this visit, as force-couple mechanics were no longer required, and the molar bands were replaced with bonded molar tubes. The archwire sequences that followed to align the labial segment were: 0.018” NiTi, 0.014 x 0.025” CuNiTi, 0.018” SS, 0.020 x 0.020” CuNiTi, and 0.019 x 0.025” SS. The archwire sequence alternated between round and rectangular NiTi and SS during the workup to the 0.019 × 0.025” SS working archwire, because the UR1 bracket was re-bonded several times to improve its position as the tooth uprighted. An elastomeric power chain was used to consolidate the labial segment, and palatal root torque was added to the working 0.019 × 0.025” SS archwire to improve the torque of the UR1. Throughout the treatment period, the patient tolerated the modified TPA and sectional fixed appliance well, with no adverse events reported. The periodontium around the maxillary incisors was healthy at the end of treatment, with probing depths of less than 3 mm and no pathological tooth mobility.

**Table 1:** De-rotation progress of the UR1.

Week (from start of active treatment)	Mechanics applied with modified TPA	Mesio-palatal rotation of UR1 (º)	De-rotation achieved since previous visit (º)
0	Force couple	110	-
4	Force couple	88	22
9	Tipping	62	26
13	Force couple	57	5
17	-	0	57

**Figure 6:**
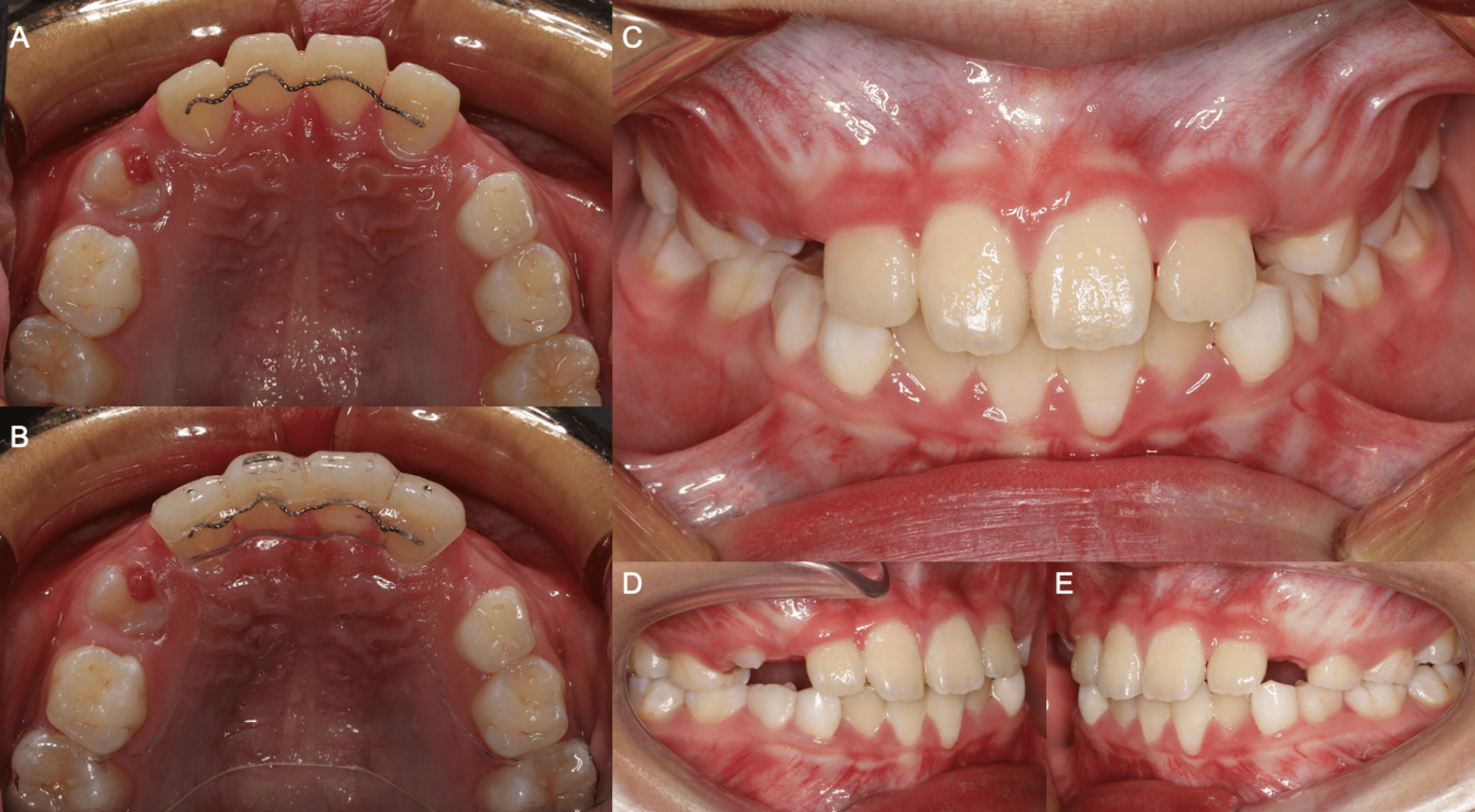
Post-treatment intra-oral photographs. (A) Occlusal view, (B) occlusal view with Theroux retainer fitted, (C) anterior occlusion, (D) right occlusion, and (E) left occlusion.

Upon removal of the fixed appliances, a lab-made fixed retainer (0.0195” SS) was bonded to the palatal surfaces of the upper incisors with composite (Transbond LR Adhesive, 3M Unitek, Monrovia, CA) using a positioning jig. A removable thermoformed Theroux retainer was provided to prevent interference with exfoliation of deciduous teeth and the eruption of permanent teeth (Figure 6). The patient was instructed to wear the retainer part-time daily (i.e., nighttime).

Prognosis

Rotated teeth are highly susceptible to relapse. Therefore, without retention strategies, the prognosis for stability would be poor [11]. Although current evidence has not yet determined the ideal retention protocol, given the high risk of relapse, a combined approach using both fixed and removable retainers was chosen for this case to maximize post-treatment stability [12].

## Discussion

**Figure 7:**
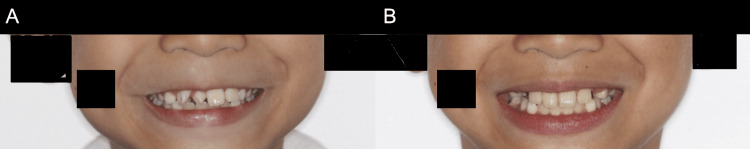
Smile aesthetics before and after interceptive treatment. (A) Pre-treatment frontal smiling view, (B) post-treatment frontal smiling view.

**Figure 8:**
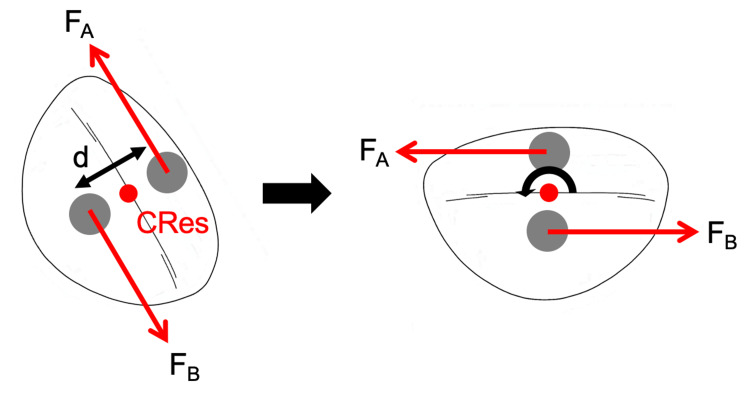
Schematic diagram demonstrating the use of a force couple to generate a counterclockwise moment to de-rotate the UR1. F_A_: force applied from the labial surface in the distal direction; F_B_: force applied from the palatal surface in the mesial direction; C_Res_: center of resistance of the tooth; d: distance separating the two forces. Image credit: Jed Lee.

This case demonstrates the successful 110° de-rotation of a maxillary incisor using a modified transpalatal arch designed to deliver a force couple (Figure 7). By incorporating extended hooks on opposite sides of the incisor, a moment was able to be generated to achieve de-rotation while minimizing undesirable movements such as tipping, intrusion, or extrusion (Figure 8). This configuration enabled a more predictable and efficient correction compared to the sole use of a sectional fixed appliance, which may have resulted in excessive proclination and compromised the periodontal health and gingival aesthetics of the UR1. From an anchorage perspective, the rigid TPA provided effective resistance to reactionary forces, with no adverse effects observed posteriorly, such as molar rotation, torque discrepancies, or vertical displacement.

An alternative method for de-rotating incisors using the whip appliance has been described in case reports by Jahanbin et al. and Parisay et al., which utilizes a removable appliance for anchorage control in combination with springs and elastics [13,14]. This appliance features a whip spring constructed from 0.4 mm stainless steel wire, which attaches from a molar tube bonded to the labial surface of the rotated maxillary incisor and to the bridge portion of the Adam’s clasp of an upper removable appliance, which the patient wears full-time. A similar method described in a case report by Sidiq et al. involves the use of intraoral elastics, which the patient attaches from buttons bonded to the rotated maxillary incisor to the clasps of an upper removable appliance [15]. Though these methods facilitate de-rotation of maxillary incisors, achieving complete alignment of UR1 in this case would remain challenging due to the significant mesial bodily movement and root torque required to align the UR1. Crucially, the effectiveness of any modality that involves the use of removable appliances will be limited by patient compliance, and research has shown that compliance with removable orthodontic appliances is often suboptimal, with patients frequently overestimating their actual wear time [16].

The advantages of the modified TPA lie in its simple, fixed design and ease of activation. This case also highlights the expanded clinical versatility of the transpalatal arch beyond its conventional role as a passive anchorage device. However, the distal-facing hooks of the modified TPA may cause discomfort and, therefore, require regular inspection to prevent soft tissue irritation or damage. As a single case report, these findings should be interpreted with caution. Future investigations, such as finite element analyses, longitudinal studies, or controlled clinical trials, are recommended to further evaluate the efficiency and potential side effects of this appliance compared with other de-rotation techniques.

## Conclusions

The modified transpalatal arch provides a simple, fixed, and effective compliance-free approach for correcting severe incisor rotations while preserving periodontal health and aesthetics. It is particularly advantageous in the mixed dentition, offering a viable alternative to removable appliances in cases where patient cooperation may be limited.
